# Design, Synthesis, and Investigation of the Biological Activities of New Thiadiazole Derivatives With Anticancer Effects

**DOI:** 10.1111/cbdd.70398

**Published:** 2026-09-14

**Authors:** Yusuf Özkay, Enes Bal, Berkant Kurban, Derya Osmaniye, Begüm Nurpelin Sağlık Özkan, Zafer Asım Kaplancıklı

**Affiliations:** ^1^ Department of Pharmaceutical Chemistry, Faculty of Pharmacy Anadolu University Eskişehir Türkiye; ^2^ Central Research Laboratory (MERLAB), Faculty of Pharmacy Anadolu University Eskişehir Türkiye; ^3^ İstanbul Süreyyapaşa Chest Diseases Training and Research Hospital İstanbul Türkiye; ^4^ Department of Pharmaceutical Chemistry, Faculty of Pharmacy Afyonkarahisar Health Sciences University Afyonkarahisar Türkiye; ^5^ Department of Pharmaceutical Chemistry, The Institute of Graduate Education Anadolu University Eskişehir Türkiye; ^6^ Pharmacy Services, Vocational School of Health Services Bilecik Şeyh Edebali University Bilecik Türkiye

**Keywords:** breast cancer, molecular docking, molecular dynamics, thiadiazole

## Abstract

Breast cancer is one of the most significant diseases in today's world. Hormone‐dependent breast cancers constitute a significant portion of breast cancers. A series of thiadiazole derivative compounds have been designed to provide a rational alternative to aromatase inhibitors, which are actively used in related cancer treatments. The designed compounds were synthesized in three steps, and their structures were elucidated using ^1^H‐NMR, ^13^C‐NMR, and HRMS spectroscopic methods. The inhibitory potentials of the obtained compounds were investigated using in vitro and in silico methods. In the studies conducted, compound **3c** stood out with its scores and values. Compound **3c** demonstrated inhibitory activity with an IC_50_ of 4.896 ± 0.210 μM against the tested cell line and an IC_50_ of 0.078 ± 0.003 μM against aromatase.

## Graphical abstract

In silico studies reveal that compound **3c** interacts strongly with the active site of the aromatase enzyme. In addition, compound **3c** yielded promising results in in vitro enzyme inhibition and cytotoxicity studies.

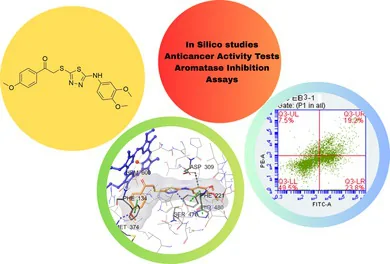

## 1 Introduction

Breast cancer is one of the most common types of cancer today but also remains a major public health threat due to its high mortality rate (Lainé et al. 2024; Lin et al. 2025). HR+/HER2− advanced BC, which constitutes approximately 60%–70% of metastatic BC cases, is a significant factor in the increasing clinical patient load. Although recurrent metastatic BC is diagnosed in 6%–10% of BC patients, 30% of cases initially diagnosed at an early stage will progress to the metastatic stage. Due to its efficacy and safety profile, ET is the fundamental treatment for HR+/HER2− advanced BC patients. Recent clinical studies on HR‐advanced BC patients have determined that extending the duration of hormone‐based treatments, such as AIs, SERDs (Selective Estrogen Receptor Degraders), and SERMs (Selective Estrogen Receptor Modulators), is of significant importance (Sánchez‐Bayona et al. 2024; Li, Deng, et al. 2024; Lu et al. 2024). Systemic chemotherapy, local radiotherapy, endocrine therapy (ET), targeted therapy, immunotherapy, and other treatment modalities constitute the nonthiasurgical approaches. BC is a disease with low predictability based on standard histopathological classifications. Despite the comprehensive application of chemotherapeutic agents and targeted drugs, recurrence and tumor resistance persist in certain BC types (Wen et al. 2024).

Aromatase (CYP19A1) is the sole enzyme limiting the conversion of androgens to estrogens. Follicle‐stimulating hormone (FSH) is the primary aromatase inducer in ovarian granulosa cells. AIs, which are capable of inhibiting estrogen biosynthesis, are highly effective in assisted reproductive technologies and the treatment of estrogen disorders. AIs inhibit estrogen synthesis without leading to a reduction in estrogen receptors. Due to their short half‐life, AIs are excreted rapidly without causing downregulation of estrogen receptors (Shi et al. 2024; Yasin et al. 2024).

Although adjuvant hormone therapy is recommended for BC patients for a duration of 5–10 years, many patients discontinue treatment early, resulting in lower survival rates. Although attention is paid to patient‐dependent reasons regarding the discontinuation of adjuvant hormone therapy, research focusing on family‐related reasons remains limited. Consequently, the survival outcomes of BC patients who discontinue adjuvant therapy due to these reasons are unknown. Estrogen exhibits cardiovascular protective effects by preventing atherosclerosis through various mechanisms, such as vasodilation of blood vessels and inhibition of vascular damage. Clinical studies have observed an increased risk of hyperlipidemia and hypertension with long‐term aromatase inhibitor (AI) use. Considering the increasing number of premenopausal and postmenopausal women using AIs for 5–10 years, the impact of long‐term AI use on cardiovascular risk and blood vessels is of vital importance for cancer survivors (Shaaban et al. 2024; Zeng et al. 2024; Gao et al. 2024).

The administration of AIs provides substantial clinical benefits for postmenopausal women diagnosed with ER+ breast cancer. Nevertheless, prolonged use of these agents frequently induces adverse musculoskeletal events, including accelerated bone density loss, muscle aches, and joint pain. This specific cluster of side effects, clinically identified as AI‐induced musculoskeletal syndrome (AIMSS), significantly compromises patient compliance and treatment continuation. Typically presenting as bilateral joint stiffness and tendinopathy—predominantly localizing in the wrists and hands—AIMSS is reported to affect approximately half of the patient population undergoing this therapeutic regimen. Normally, while primary well‐differentiated tumors are sensitive to endocrine therapy, their hormone sensitivity may diminish over time following adjuvant treatments such as radiotherapy and/or chemotherapy (Luijten et al. 2024; Holt et al. 2024).

Compounds derived from 1,3,4‐thiadiazole, which possess anti‐inflammatory, antibacterial, antiviral, antihypertensive, and anticancer effects, have been extensively studied using in vitro and in silico methods due to their inhibitory potential (Mostefai et al. 2025; Li, Liu, et al. 2024; Gomha, Abdelhamid, et al. 2018; Gomha, Muhammad, and El‐Reedy 2018; El‐Enany et al. 2021, 2020; Gomha et al. 2019; Elroby et al. 2024; Said et al. 2022). In addition, in recent years, numerous studies have been conducted on the aromatase inhibitor potential of thiadiazole derivatives (Rashdan et al. 2024; Mayhoub et al. 2012; Demiraran et al. 2024).

In light of all this information, 10 different thiadiazoles with high affinity for the aromatase enzyme were synthesized for the discovery of selective anticancer agents. The structures of the compounds obtained were elucidated and their biological activities were investigated.

## 2 Results and Discussion

### 2.1 Chemistry

**SCHEME 1:**
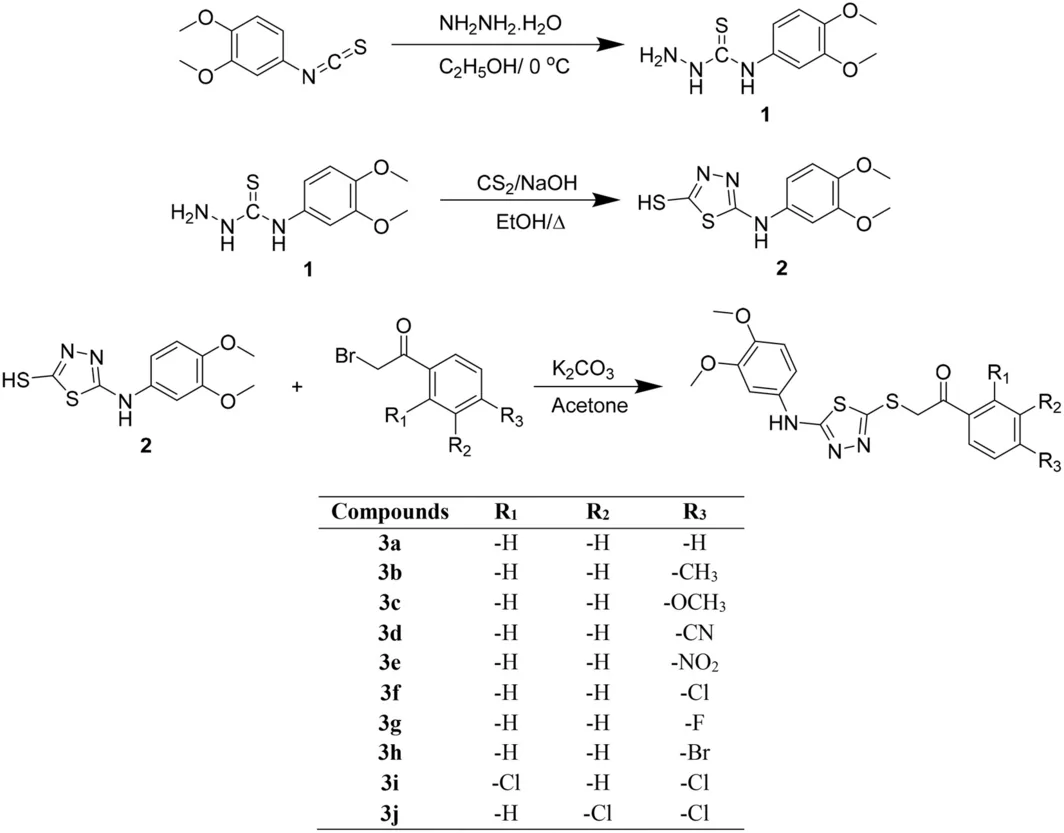
Synthesis pathway for obtained compounds (**3a–3j**).

Compounds **3a–3j** were synthesized in three steps as shown in Scheme 1. In the first step, a thiosemicarbazide structure was obtained from 3,4‐dimethoxyphenyl isothiocyanate. Subsequently, the thiadiazole ring was closed via the thiosemicarbazide. In the final step, 10 different thiadiazole derivative compounds were obtained by substitution of appropriate phenacyl bromides into the structure. The structures of the compounds obtained were established by spectroscopic methods, namely ^1^H‐NMR, ^13^C‐NMR, and HRMS (Data S1). The spectral data consistently support the proposed structures and highlight the targeted functional group modifications. In the ^1^H‐NMR spectra, the presence of the 3,4‐dimethoxyphenyl group was confirmed by the methoxy (–OCH_3_) protons resonating at 3.72 and 3.74–3.76 ppm. The methylene (–CH_2_–) protons of the thioether linkage appeared between 4.78 and 5.00 ppm, while the secondary amine (–NH) protons were recorded downfield at 10.20–10.29 ppm. Specific modifications on the acetophenone ring were also distinctly identifiable. For instance, the methyl protons of compound **3b** were observed at 2.40 ppm, and the additional methoxy protons of compound **3c** appeared at 3.87 ppm. The ^13^C‐NMR data further validated the core scaffold, with the aliphatic methylene carbons appearing at 41.75–44.00 ppm and the dimethoxyphenyl methoxy carbons at 55.90–56.37 ppm. The characteristic carbonyl (C=O) carbons were recorded in the expected downfield region at 192.05–194.86 ppm, alongside the thiadiazole carbons which resonated around 149.47–151.49 and 166.01–166.20 ppm. Finally, the exact mass measurements obtained via HRMS analysis were in tight agreement with the calculated values for all synthesized derivatives, confirming their elemental compositions.

### 2.2 Anticancer Activity Tests

#### 2.2.1 Cytotoxicity Assay

The activities of the 10 compounds and doxorubicin against the A‐549, MCF‐7, and NIH‐3T3 cell lines are presented in Table 1. Upon reviewing the relevant data, the IC_50_ values of compounds **3a**, **3b**, **3c**, and **3f** were found to be lower than 10 μM, and aromatase enzyme inhibition assays were performed on these compounds. Furthermore, the anticancer activity of compound **3c**, which stands out with its IC_50_ value of 4.896 ± 0.210 μM, was further investigated using flow cytometry analysis.

**TABLE 1:** IC_50_ values (μM) of compounds (**3a–3j**) against A549, MCF‐7, and NIH/3T3 cell lines.

Compounds	A‐549	MCF‐7	NIH/3T3
**3a**	45.130 ± 1.530	**6.045 ± 0.278**	> 100
**3b**	> 100	**7.104 ± 0.301**	55.411 ± 2.041
**3c**	27.580 ± 1.104	**4.896 ± 0.210**	> 100
**3d**	> 100	> 100	> 100
**3e**	> 100	> 100	> 100
**3f**	> 100	**9.021 ± 0.399**	41.158 ± 1.845
**3g**	36.455 ± 1.085	13.024 ± 0.578	69.023 ± 2.345
**3h**	> 100	> 100	> 100
**3i**	31.855 ± 1.123	22.475 ± 0.974	> 100
**3j**	> 100	14.654 ± 0.664	80.664 ± 3.104
Doxorubicin	10.985 ± 0.247	1.940 ± 0.084	> 100

*Note:* Bold values represent the compounds exhibiting an IC_50_ value of < 10 μM against the MCF‐7 cell line.

When the activities of the compounds against the A‐549 cell line were examined, it was observed that the IC_50_ values of compound **3a**, compound **3c**, compound **3g**, and compound **3i** ranged from 27.580 ± 1.104 to 45.130 ± 1.530 μM. In addition, no significant activity against the A‐549 cell line was observed for the other compounds.

The fact that compound **3a** (IC_50_ = 6.045 ± 0.278 μM) and compound **3c** (IC_50_ = 4.896 ± 0.210 μM), which exhibited the highest activity against the MCF‐7 cell line, did not show activity against the NIH‐3T3 cell line (healthy mouse embryonic fibroblast cell line) was interpreted positively. Additionally, compound **3f** exhibited the highest activity against the NIH‐3T3 cell line with an IC_50_ value of 41.158 ± 1.845 μM.

A structure–activity relationship (SAR) analysis was conducted to evaluate the influence of the substituents (R) on the phenyl ring. The observed biological activity trends strongly correlate with the molecular docking results, which reveal that this region of the molecule occupies a hydrophobic pocket within the enzyme's active site. The highest activity was observed for the unsubstituted derivative (R=H) and compounds bearing lipophilic, electron‐donating groups (R=CH_3_ and OCH_3_), as these substituents favorably interact with the non‐polar residues of the pocket. Halogen substitution (R=F, Cl, and dichloro) led to a moderate decrease in activity; although these atoms are lipophilic, their fit within the binding site is less optimal than that of the methyl or methoxy groups. Conversely, the introduction of highly polar, strong electron‐withdrawing groups (R=NO_2_, CN) resulted in the lowest activity. This dramatic drop is likely due to an energetic penalty associated with forcing highly polar moieties into a hydrophobic environment. Additionally, the poor activity of the bromo‐substituted derivative (R=Br) points to a strict volume limitation within the pocket, suggesting that bulkier halogens cause steric clashes. These combined findings demonstrate that both the lipophilic nature and the steric boundaries of the enzyme's pocket are the primary drivers of biological activity for this compound series.

#### 2.2.2 Flow Cytometry Analysis

A protein called Annexin V has the ability to bind to phosphatidylserine and, when conjugated with fluorescent dyes, enable the use of flow cytometry to identify apoptotic cells. When Annexin V‐FITC and EI are used as fluorescent chemicals, cells are categorized into quadrants (necrosis, early apoptosis, and late apoptosis) according to the complexes they form with the corresponding dyes, as assessed by flow cytometry analysis. Late apoptotic cells bind to both substances in the upper right quadrant; early apoptotic cells only bind to Annexin V in the lower right quadrant; necrotic cells only bind to EI in the upper left quadrant; and healthy cells do not bind to either substance in the lower left quadrant (Hidir et al. 2025).

**FIGURE 1:**
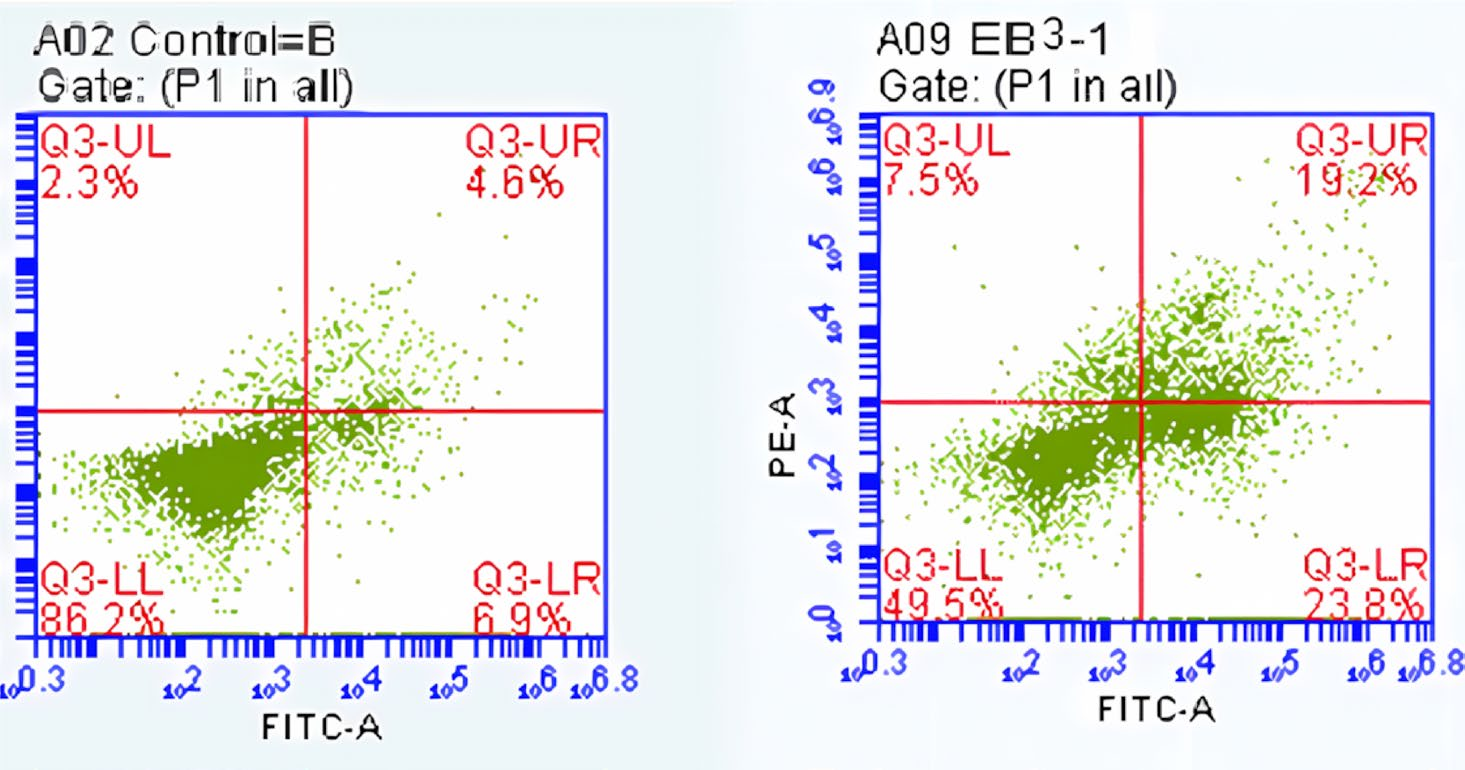
Flow cytometric analysis diagram of compounds **3c** in the MCF‐7 cell line.

As shown in Figure 1, compound **3c** induced necrosis in 7.52% of the MCF‐7 cell line, while driving 19.2% into late apoptosis and 23.8% into early apoptosis. Due to its non‐dominant necrotic behavior, compound **3c** is considered to have the potential to act as a non‐cytotoxic anticancer agent.

### 2.3 Aromatase Inhibition Assay

Compounds **3a**, **3b**, **3c**, and **3f**, which exhibited the highest activity against the MCF‐7 cell line in MTT cytotoxicity assays and had IC_50_ values below 10 μM, were included in the aromatase enzyme inhibition assays. Letrozole was used as the reference compound in these tests. As a result of these experiments, compound **3c** was the compound with the closest values to the reference compound letrozole, with an IC_50_ value of 0.078 ± 0.003 μM. In addition, compounds **3a**, **3b**, and **3f** had IC_50_ values of 0.113 ± 0.004, 0.233 ± 0.010, and 0.172 ± 0.007 μM, respectively, against the aromatase enzyme (Table 2).

**TABLE 2:** IC_50_ values (μM) of compounds **3a, 3b, 3c**, **3f**, and letrozole.

Compounds	Aromatase inhibition
**3a**	0.113 ± 0.004
**3b**	0.233 ± 0.010
**3c**	0.078 ± 0.003
**3f**	0.172 ± 0.007
Letrozole	0.031 ± 0.001

### 2.4 Molecular Docking

**FIGURE 2:**
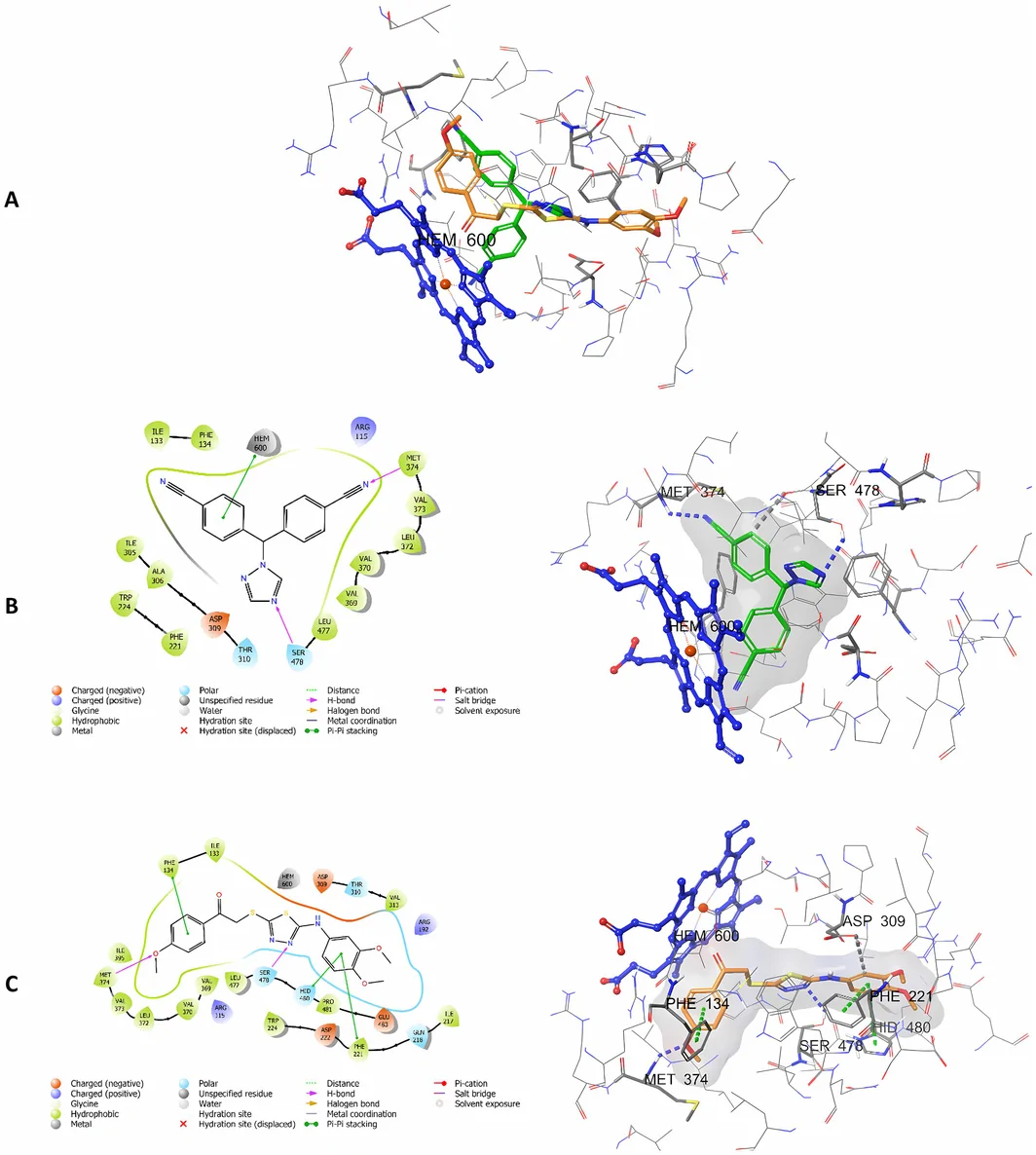
(A) Overlay of the binding poses of compound **3c** (orange) and letrozole (green) with the aromatase enzyme. Two‐dimensional and three‐dimensional interaction profiles of letrozole (B) and derivative **3c** (C) with the active site of aromatase (PDB ID: 3EQM).

To ensure the reliability of the docking environment, a protocol validation was conducted by re‐docking the native ligand into the 3EQM binding pocket, achieving a Root Mean Square Deviation (RMSD) of 0.85 Å. The receptor grid was centered on the native ligand within a 20 × 20 × 20 Å cubic box. Both protein and ligands were prepared at physiological pH 7.4, with appropriate protonation and tautomeric states assigned. For comparative analysis, the reference drug letrozole was docked using the same parameters, showing a binding energy and interaction profile consistent with the experimental potency of compound **3c**. Comparative docking analysis revealed that compound **3c** and letrozole adopt a very similar binding orientation within the aromatase active site. Both molecules demonstrate a consistent spatial alignment and share a similar interaction network, particularly involving interactions with Met374 and Ser 478 (Figure 2). This high degree of positional and functional overlap with a clinically used inhibitor further validates the potent aromatase inhibition potential of the synthesized compound.

**FIGURE 3:**
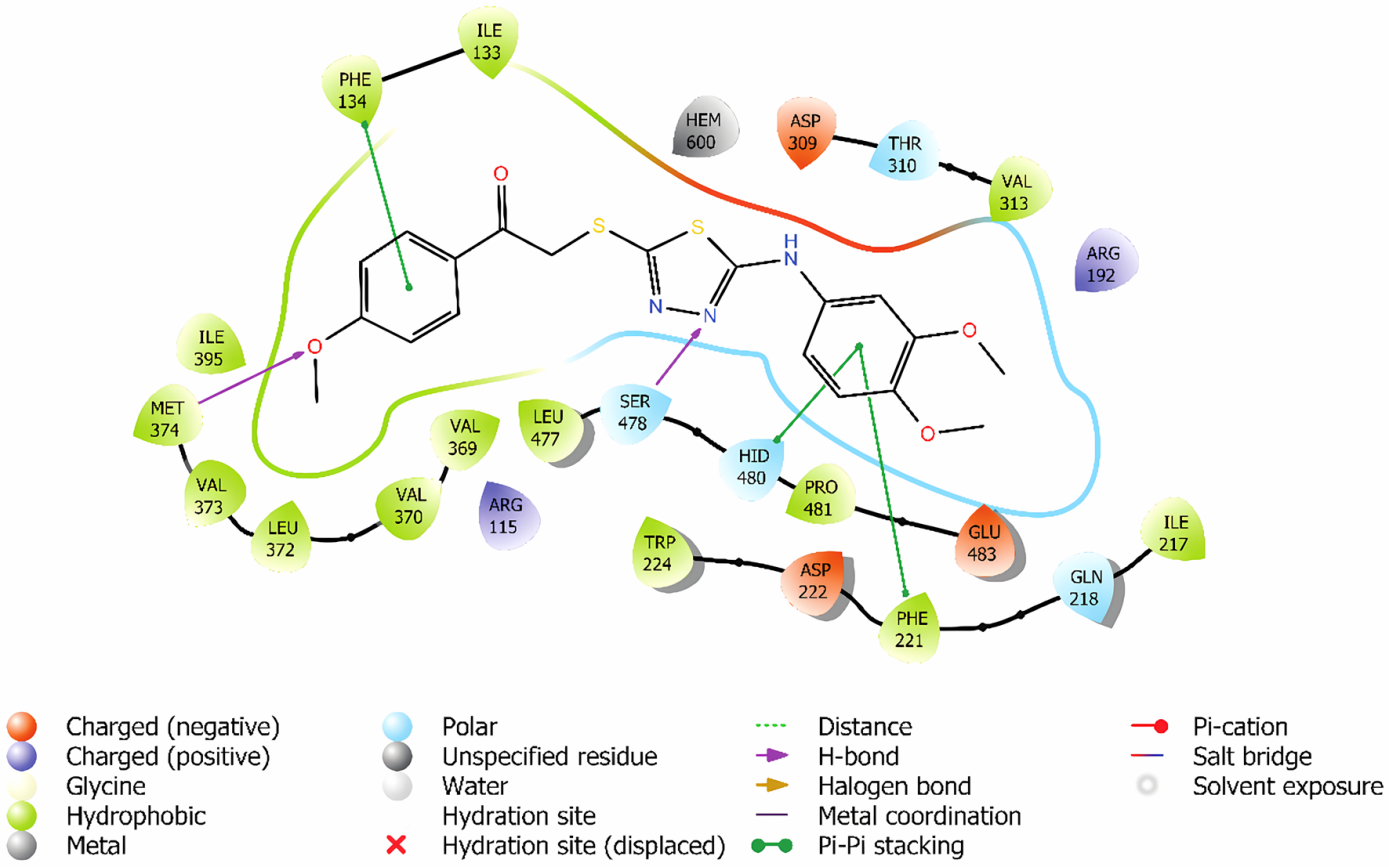
2D interaction of compound **3c** at binding region (PBDID: 3EQM).

**FIGURE 4:**
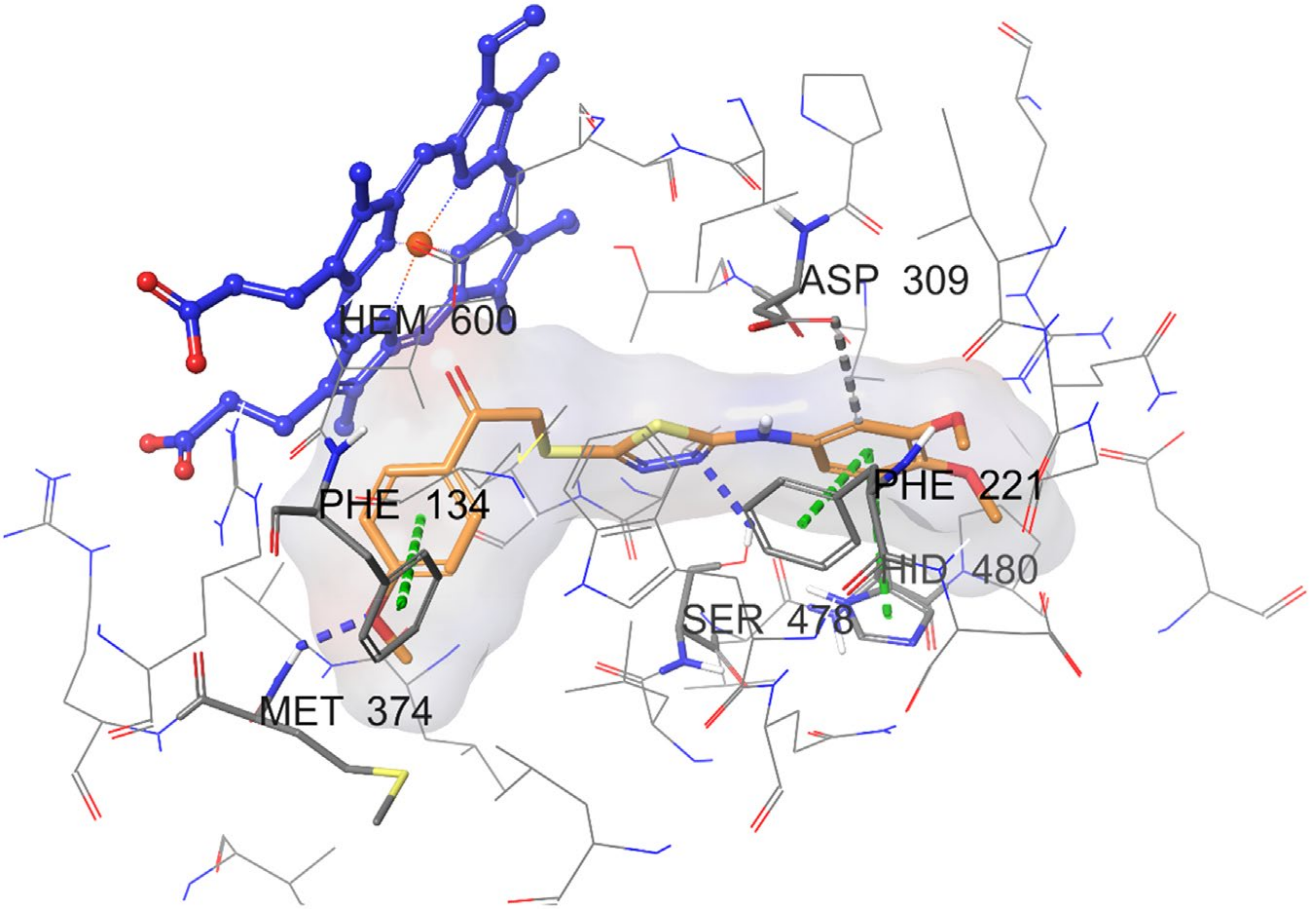
The three‐dimensional interacting mode of compound **3c** in the active region of aromatase enzyme (PDB ID: 3EQM).

According to Figure 2, three different π–π bonds and two different H‐Bonds were observed between compound **3c** and the 3EQM crystal. A π–π interaction was observed between the 1,3,4‐disubstituted benzene moiety of compound **3c** and the Phe 134 residue of the enzyme. Two different π–π bonds were also observed between the 1,3,4‐trisubstituted benzene moiety of compound **3c** and the enzyme's Phe 221 and Hid 480 residues. Two different H‐bond interactions with the enzyme were observed, one via the thiadiazole moiety and one via the methoxy moiety of compound **3c** (Figures 3 and 4).

### 2.5 Molecular Dynamics Simulation

To investigate the dynamic stability of the **3c**‐3EQM complex, a 100 ns MD simulation was executed. The system was prepared using the System Builder tool with the OPLS4 force field and TIP3P water model in an orthorhombic box (10 Å buffer). Physiological neutrality was ensured by adding 0.15 M NaCl ions. The simulation utilized a multi‐stage equilibration including NVT and NPT ensembles with positional restraints on protein C‐alpha atoms during the initial phases. The binding stability was quantified by monitoring the Heme iron (Fe) coordination, which maintained a 91% occupancy rate defined by a 2.5 Å distance threshold throughout the 100 ns production run. These parameters confirm that the inhibitor remains securely anchored within the hydrophobic catalytic pocket without the requirement of a lipid bilayer environment for structural integrity. The structural integrity and binding affinity of the complex were comprehensively evaluated using various metrics, including Root Mean Square Deviation (RMSD), Root Mean Square Fluctuation (RMSF), and ligand‐specific physicochemical parameters (Table 3).

**TABLE 3:** RMSD, RMSF and Rg parameters (Å) for compound **3c** on aromatase enzyme.

Complex	RMSD	Rg	RMSF
**3c‐3EQM**	2.4 Å	5.25–5.50 Å	Arg115 (0.49 Å), Ile133 (0.58 Å), Phe134 (0.60 Å), Leu188 (0.61 Å), Arg192 (0.53 Å), Ile217 (1.49 Å), Gln218 (1.46 Å), Phe221 (0.89 Å), Trp224 (0.72 Å), Ile305 (0.69 Å), Ala306 (0.67 Å), Asp309 (0.56 Å), Val313 (0.50 Å), Val369 (0.53 Å), Val370 (0.53 Å), Leu372 (0.53 Å), Met374 (0.45 Å), Cys437 (0.46 Å), Leu477 (0.81 Å), Ser478 (0.62 Å), Leu479 (0.64 Å), His480 (0.77 Å), Pro481 (0.86 Å), Glu483 (1.19 Å)

**FIGURE 5:**
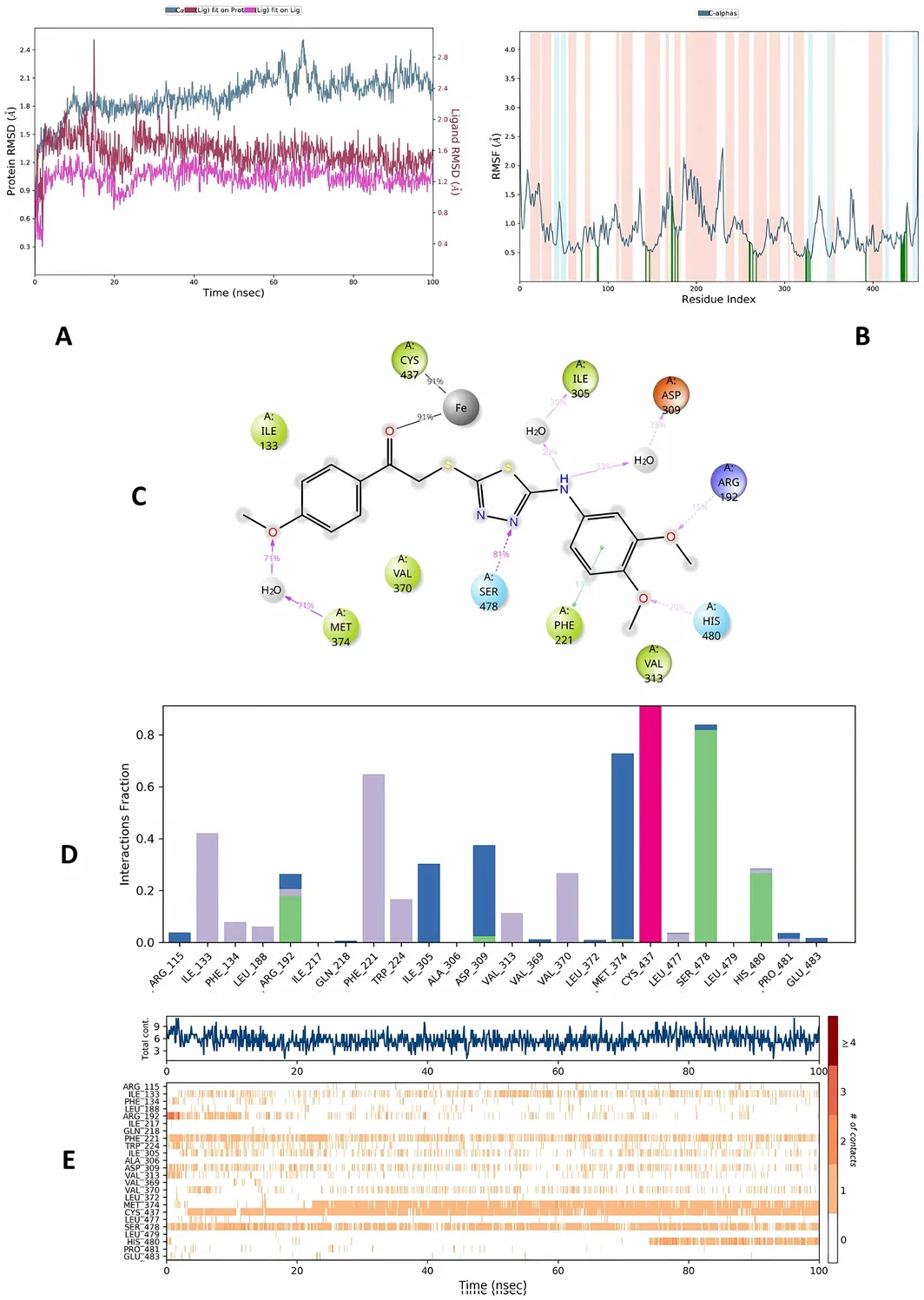
Molecular dynamic results (A–E) of compound **3c‐3EQM** complex.

To evaluate the dynamic stability of the simulated system, we first monitored the root‐mean‐square deviation (RMSD) for both the ligand's heavy atoms and the enzyme's alpha‐carbon (Cα) backbone across the simulation timeframe, referenced against their starting coordinates (Figure 5A). As illustrated by the blue trajectory, the backbone RMSD experienced a steady climb over the first 40 ns, maxing out near 2.4 Å. This initial fluctuation corresponds to the structural adaptation and relaxation of the protein around the bound inhibitor. After this adaptation period, the complex achieved a state of dynamic equilibrium, as evidenced by the RMSD values stabilizing and consistently fluctuating within a narrow 2.1–2.4 Å window for the rest of the simulation. Simultaneously, the RMSD of compound **3c** (red line), calculated by fitting the ligand to the protein backbone, remained highly consistent with the protein's stability, fluctuating within a narrow range of 1.6–1.8 Å. This stable trajectory confirms that the ligand remained securely anchored within the binding pocket without undergoing significant translational or rotational shifts.

The local flexibility of the protein residues and the stabilizing effect of ligand binding were analyzed through the RMSF profile (Figure 5B). The simulation results revealed that the secondary structural elements (α‐helices and β‐sheets) maintained their rigidity with fluctuations remaining well below 1.5 Å. The highest fluctuations were observed in the flexible loop regions, particularly around residue indices 170–200 and 220–250. Notably, the residues directly involved in ligand binding, such as Arg192, Ile305, Met374, Cys437, and Ser478 (highlighted with green bars), exhibited remarkably low RMSF values (typically < 0.8 Å). This localized stabilization indicates that compound **3c** effectively restricts the mobility of these critical residues, thereby strengthening the enzyme‐inhibitor complex.

The specific molecular interactions governing the high binding affinity of compound **3c** were examined using the 2D interaction diagram (Figure 5C). A key finding of the simulation is the exceptionally high occupancy of the metal coordination between the carbonyl group of the ligand and the Heme iron (Fe) at the catalytic center, maintained at a fraction of 91%. This interaction is crucial for the direct inhibition of the aromatase enzyme's catalytic activity. Furthermore, the ligand established a robust hydrogen bonding network, including a direct hydrogen bond with Ser478 (%81) and significant water‐bridged interactions with Met374 (%71), Asp309 (%33), and Ile305 (%30). Hydrophobic contributions and pi‐pi stacking interactions with Phe221 (%13) further stabilized the aromatic moieties of the ligand within the hydrophobic core of the active site, while ionic/polar contacts with Arg192 and His480 completed the binding envelope (Table 4).

**TABLE 4:** Interacting amino acids and fractions for compound **3c** on aromatase enzyme.

Complex	Amino acids interacting above 10%	Interaction fractions
**3c‐3EQM**	Arg192 (H‐ bond: 15%) Phe221 (π–π interaction: 13%) Ile305 (Water mediated H‐bond: 30%) Asp309 (Water mediated H‐bond: 33%) Met374 (Water mediated H‐bond: 71%) Ser478 (H‐ bond: 81%) His480 (H‐ bond: 20%) HEM (Metal coordination 91%)	*Water mediated H‐bond (blue)* Arg115, Arg192, Gln218, Ile305, Ala306, Asp309, Val369, Leu372, Met374, Leu477, Ser478, Leu479, His480, Pro481, Glu483
		*H‐bond (green)* Arg192, Asp309, Met374, Ser478, His480
		*Ionic interaction (pink)* Cys437
		*Hydrophobic interaction (purple)* Ile133, Phe134, Leu188, Arg192, Phe221, Trp224, Val313, Val370, Met374, Leu477, His480, Pro481

The quantitative distribution and the types of these interactions were further validated by the interaction fractions histogram (Figure 5D). The analysis confirms that the binding is driven by a synergistic combination of hydrogen bonding, hydrophobic contacts, and water bridges. Ser478 emerged as the primary contributor to the hydrogen bonding network with a fraction exceeding 0.8, whereas Phe221 and Ile133 provided substantial hydrophobic stabilization. The presence of significant water‐mediated bridges with Met374 and Asp309 highlights the role of the solvent environment in mediating and stabilizing the ligand‐enzyme interface, ensuring the persistence of the binding mode throughout the dynamic process.

The temporal stability and continuity of the protein‐ligand contacts were visualized in the interaction timeline (Figure 5E). The top panel shows that the total number of contacts fluctuated between 6 and 9 throughout the 100 ns simulation, indicating a high level of interaction saturation. The bottom panel demonstrates that the interactions with key residues, specifically Phe221, Cys437, and Ser478, were nearly constant, represented by dark, continuous color intensities. The interaction with Met374 became particularly prominent after the first 25 ns and remained stable until the end of the simulation. This sustained contact profile suggests that the binding of compound **3c** is not merely transient but kinetically stable, which is a desirable trait for effective enzyme inhibition.

**FIGURE 6:**
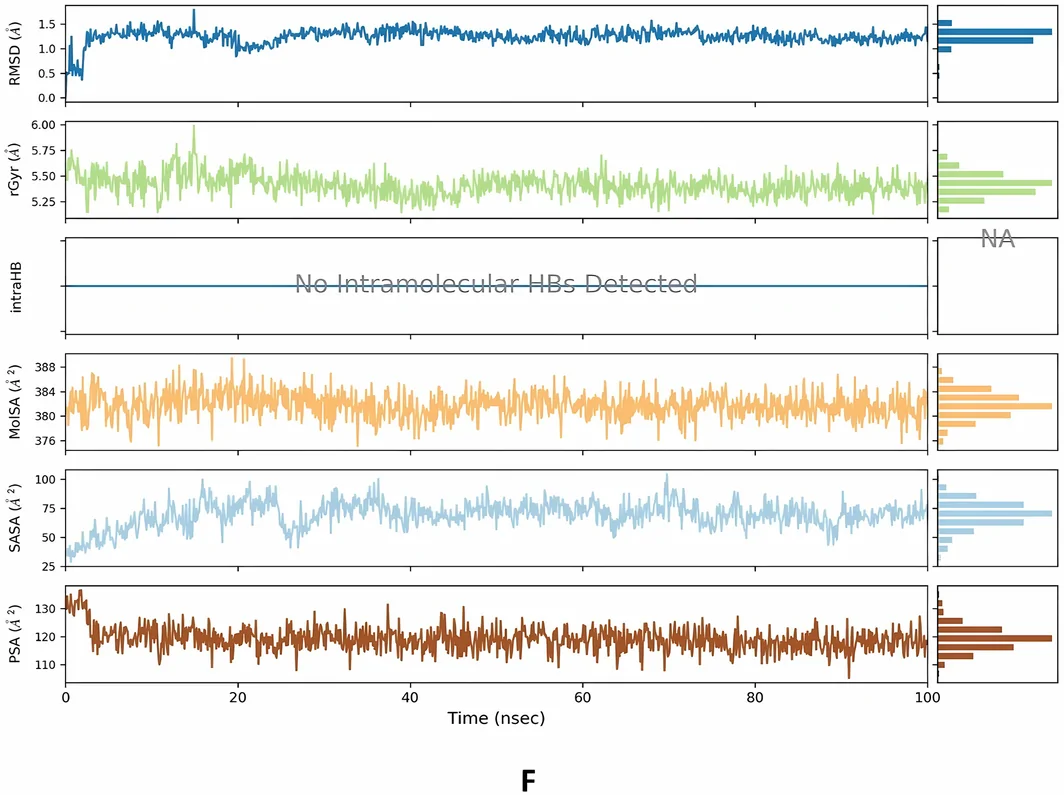
Molecular dynamic results (**F**) of compound **3c‐3EQM** complex.

Finally, the physicochemical properties and conformational compactness of compound **3c** were monitored via ligand property analyses (Figure 6F). The internal RMSD of the ligand (top panel) remained remarkably low at approximately 1.2 Å, confirming that the molecule did not undergo significant internal strain or distortion. The Radius of Gyration (rGyr) maintained a stable profile between 5.25 and 5.50 Å, indicating that the ligand retained a compact conformation within the catalytic pocket. Other metrics, such as Molecular Surface Area (MolSA), Solvent Accessible Surface Area (SASA), and Polar Surface Area (PSA), showed consistent values throughout the 100 ns period, reflecting an energetically favorable and stable binding orientation.

In conclusion, these MD results provide strong computational evidence that compound **3c** is a potent aromatase inhibitor that forms a highly stable and structurally optimized complex with the aromatase enzyme (3EQM crystal).

## 3 Conclusion

The anticancer effects of the compounds obtained on breast and lung cancer cells were investigated in healthy mouse fibroblast cells using the reference drug Doxorubicin. When the cytotoxic effect of the original compounds on the MCF‐7 cell line was evaluated, compounds **3a**, **3b**, **3c**, and **3f** were found to have significant anticancer effects among the compounds obtained. In in vitro studies, compound **3c** (IC_50_ = 4.896 ± 0.210 μM) in particular showed anticancer effects close to those of the reference compound Doxorubicin (IC_50_ = 1.940 ± 0.084 μM). Due to their anticancer activity, these compounds were selected for aromatase inhibition studies. The reference drug Letrozole has an IC_50_ of 0.031 ± 0.001 μM. Among the tested derivatives, compound **3c** exhibited the most potent aromatase inhibitory activity with an IC_50_ of 0.078 ± 0.003 μM, followed by compound **3a** (0.113 ± 0.004 μM), compound **3f** (0.172 ± 0.007 μM), and compound **3b** (0.233 ± 0.010 μM). This strong correlation suggests that the notable MCF‐7 cytotoxicity of compound **3c** is closely associated with its robust aromatase inhibitory potential. However, while aromatase inhibition likely plays a primary role, the induction of apoptosis may also be mediated by additional complementary intracellular mechanisms. Furthermore, compound **3c** exhibited low cytotoxicity against the murine NIH‐3T3 fibroblast cell line. While this finding points towards a preliminary and favorable selectivity profile in vitro, we acknowledge that NIH‐3T3 is of murine origin. Therefore, to make definitive claims regarding human safety and tumor selectivity, further toxicity studies utilizing normal human cell lines are necessary. To determine whether compound **3c** exerted its effect via the apoptotic or necrotic pathway in the MCF‐7 cell line, flow cytometry was performed using the Annexin V/EIA method. At the IC_50_ concentration of compound **3c**, the necrosis rate was 7.52%, the late apoptosis rate was 19.17%, and the early apoptosis rate was 23.81%.

Compound **3c** showed promising results in in vitro studies, so a in silico studies were conducted between compound **3c** and the active site of the aromatase enzyme (PDB ID: 3EQM). In silico studies reveal that compound **3c** interacts strongly with the active site of the aromatase enzyme and that this interaction supports biological activity. This indicates that compound **3c** is effective in inhibiting the aromatase enzyme.

The findings offer promising results for cancer treatment. Therefore, it is anticipated that designing anticancer compounds with similar structures could pave the way for a radical approach in cancer treatment.

## 4 Experimental

### 4.1 Chemistry

Commercially available chemical reagents and solvents were utilized directly without any prior purification steps. Uncorrected melting point values were recorded utilizing a Mettler Toledo‐MP90 apparatus. Nuclear magnetic resonance spectra (^1^H and ^13^C) were acquired on a Bruker DPX FT‐NMR spectrometer operating at 300 and 75 MHz, respectively. High‐resolution mass spectrometry (HRMS) data were obtained via an electrospray ionization (ESI) source on a Shimadzu LCMS‐IT‐TOF system.

#### 4.1.1 Synthesis of N‐(3,4‐Dimetoxyphenyl)hydrazinecarbothioamide (**1**)

A solution of 3,4‐dimethoxyphenyl isothiocyanate (5.0 g, 25.6 mmol) in ethanol was cooled in an ice bath, followed by the dropwise addition of hydrazine hydrate (3.08 mL, 62.77 mmol). The progress of the reaction mixture was tracked using thin‐layer chromatography (TLC). Once complete, the formed solid was collected via filtration, rinsed with distilled water, dried, and subsequently recrystallized from ethanol.

#### 4.1.2 Synthesis of 5‐((3,4‐Dimetoxyphenyl)amino)‐1,3,4‐Thiadiazole‐2‐Thiol (**2**)

*N*‐(3,4‐dimethoxyphenyl)hydrazinecarbotihoamide (**1**) (5.41 g, 23.83 mmol) was dissolved in ethanol. To this mixture, carbon disulfide (1.71 mL, 28.49 mmol) and sodium hydroxide (0.95 g, 23.83 mmol) were added. The reaction mixture was heated under reflux and reaction progress was monitored by TLC. Upon completion of the reaction, the mixture was cooled to room temperature and ice was added. The pH was adjusted to 2 using 20% HCl. The resulting precipitate was filtered, washed with distilled water, and recrystallized from ethanol.

#### 4.1.3 Synthesis of the Target Compounds (**3a–3j**)

To synthesize the final derivatives (**3a–3j**), an equimolar mixture of the thiadiazole‐2‐thiol intermediate 2 (0.3 g, 1.12 mmol) and the corresponding substituted phenacyl bromide (1.12 mmol) was refluxed in acetone, utilizing potassium carbonate (0.154 g, 1.12 mmol) as a base. Upon reaction completion, the resulting crude solids were collected by filtration, repeatedly washed with distilled water, and ultimately purified via recrystallization from hot ethanol.

### 4.2 Anticancer Activity Tests

#### 4.2.1 Cytotoxicity Assay

The cytotoxic effects of compounds **3a**–**3j** against A‐549, MCF‐7, and NIH‐3T3 cell lines were investigated using the MTT method. Doxorubicin was used as the reference active substance during these experiments. All MTT assays were performed in four independent replicates (*n* = 4). The optical densities were measured at 540 nm using a BioTek (USA) microplate reader. Data are presented as the mean ± standard deviation (SD).

#### 4.2.2 Flow Cytometry Analysis

Compound **3c** was used in flow cytometric analyses because it was the compound that stood out in activity screening. Cells were treated with compound **3c** and the positive control, erlotinib, at their respective IC_50_ concentrations for 24 h. The “Apoptotic, Necrotic, and Healthy Cells Detection Kit” (http://www.Abpbio.Com/Product/Apoptotic‐Necrotic‐Kit/) was used to identify apoptotic and necrotic cells. Analyses were performed using a FACS Aria I Cell Sorter Flow Cytometer (Becton Dickinson, San Jose, CA, USA), and 10,000 events were recorded for each sample. Data acquisition and analysis were conducted using BD FACSDiva Software (Version 6.1.2). This included proper compensation for fluorochrome signal overlap and the application of a specific gating strategy to isolate the main cell population (parent) from debris before evaluating the percentages of cells in the four distinct apoptotic/necrotic quadrants (Hidir et al. 2025; Halimi Syla et al. 2025; Yurtdaş‐Kırımlıoğlu et al. 2020).

### 4.3 Aromatase Inhibition Assay

The Bio Vision Aromatase (CYP19A) Inhibitor Screening Kit (Fluorometric) procedure was performed. Compounds were dissolved in dimethyl sulfoxide (DMSO). As recommended by the manufacturer's protocol, the final DMSO concentration in the assay wells was strictly maintained below 0.25% (v/v) to prevent solvent‐induced aromatase inhibition. At least 7 different concentrations ranging from 10^−3^ to 10^−9^ M were added to generate multi‐point dose–response curves. A recombinant human aromatase stock was prepared using 1 mL of aromatase assay buffer. The contents were thoroughly mixed with a vortex to achieve a homogeneous solution. The mixture was transferred to a 15 mL conical tube. The volume was made up to 2450 μL with Aromatase Assay Buffer. To reach a total volume of 2.5 mL, 50 μL of NADPH Production System (100×) was added. The letrozole was selected as the reference positive control. The assay design explicitly included solvent controls (assay buffer containing DMSO without test compounds, representing 100% uninhibited enzyme activity) and background controls (reaction mixture devoid of the fluorogenic aromatase substrate) to accurately determine baseline fluorescence. To allow the test ligands to interact with aromatase, the plate was incubated at 37°C for a minimum of 10 min. At the end of the incubation period, 30 μL of Aromatase Substrate/NADP+ solution was added to each well separately. Within 1 min, the fluorescence value was measured at an excitation/emission (Ex/Em) wavelength of 488/527 nm. Finally, the percent inhibition due to the test ligands was calculated, and the IC_50_ values were determined via non‐linear regression analysis using Microsoft Excel software.

### 4.4 Molecular Docking

To elucidate the binding mechanism and predict the intermolecular interactions between the most active derivative (compound **3c**) and its biological target, structure‐based computational docking was executed. The high‐resolution X‐ray crystallographic structure of the human aromatase enzyme (PDB ID: 3EQM; 2.90 Å resolution) was utilized as the receptor model (Ghosh et al. 2009). All computational simulations were conducted within the Schrödinger Maestro suite (version 2020.2). Initial receptor refinement was carried out via the Protein Preparation Wizard, which involved optimizing bond orders, assigning appropriate protonation states, and minimizing the energy of the complex using the OPLS 2005 force field. Subsequently, the three‐dimensional conformation of the ligand was generated utilizing LigPrep, and the molecular docking simulations were performed using the Glide module under single precision (SP) parameters.

The crystal structure was prepared for docking studies using the Protein Preparation Wizard (V. Maestro, 10.6; Schrödinger LLC, New York, NY, USA 2016) module. The aromatase enzyme was minimized using the OPLS 2005 force field, bond lengths were optimized, and the potential charges that atoms on charged amino acids could acquire under the specified environment conditions were automatically calculated. For the docking process, compound **3c** was prepared using the LigPrep 3.8 module (“Schrödinger, LigPrep, Version 3.8; Schrödinger LLC, New York, NY, USA” 2020), then a grid was created using the Glide 7.1 module (L. Schrödinger, Glide, Version 7.1; Schrödinger LLC, New York, NY, USA 2016), and the docking process was performed using the single precision method. As a result, the molecular modeling studies were completed.

### 4.5 Molecular Dynamics Simulation

The dynamic behavior of the protein‐ligand complexes was simulated over a 100 ns timeframe utilizing the Desmond module of Schrödinger Maestro, implementing a POPE lipid bilayer environment to replicate physiological membrane conditions (Liu et al. 2018; “M.‐D.I. Tools, Schrödinger LLC, New York, NY, Schrödinger Release 2018‐3: Prime (2018)” 2020; Sureshkumar et al. 2018; Humphreys et al. 1994; Hoover 1985; Martyna et al. 1994; Essmann et al. 1995).

## Author Contributions

**Yusuf Özkay:** resources, writing – original draft, visualization, supervision, methodology. **Enes Bal:** data curation, conceptualization. **Berkant Kurban:** conceptualization, methodology, data curation, writing – original draft. **Derya Osmaniye:** conceptualization, methodology, software, formal analysis, investigation, resources, data curation, writing – original draft, visualization. **Begüm Nurpelin Sağlık Özkan:** software, methodology, writing – original draft. **Zafer Asım Kaplancıklı:** conceptualization, investigation, resources, writing – review and editing, visualization.

## Funding

The authors have nothing to report.

## Conflicts of Interest

The authors declare no conflicts of interest.

## Supporting information


**Data S1:** cbdd70398‐sup‐0001‐DataS1.docx.

## Data Availability

The data that supports the findings of this study are available in Supporting Information of this article.
