# Supplementary material for: Design, Synthesis, and Investigation of the Biological Activities of New Thiadiazole Derivatives With Anticancer Effects
Source: Chem Biol Drug Des. 2026 Sep 14;108(3):e70398. doi: 10.1111/cbdd.70398 (PMC13575461; doi:10.1111/cbdd.70398)
Supplement: Supplementary file 1 [file CBDD-108-e70398-s001.docx]

**SUPPORTING INFORMATION**

**Design, Synthesis, and Investigation of The Biological Activities of New Thiadiazole Derivatives With Anticancer Effects**

**1. Experimental**

1.1. Chemistry

*1.1.3. Synthesis of the target compounds (****3a-3j****)*

*1.1.3.1. 2-((5-((3,4-Dimethoxyphenyl)amino)-1,3,4-thiadiazol-2-yl)thio)-1-phenylethan-1-one (****3a****)*

Yield: 78 % (0.339 g), purity (HPLC): >99.9%, M.P.: 168.5-169.1 ^o^C. ^1^H-NMR (300 MHz, DMSO-*d_6_*): δ = 3.72 (3H, s, -OCH_3_), 3.74 (3H, s, -OCH_3_), 4.95 (2H, s, -CH_2_), 6.92 (1H, d, *J*=4.5 Hz trisubstituted benzene), 7.03 (1H, dd, *J_1_*=1.5 Hz, *J_2_*=9 Hz, trisubstituebenzene), 7.23 (1H, d, *J*= 1.5 Hz, trisubstituted benzene), 7.55-7.60 (2H, m, monosubstituted benzene), 7.67-7.73 (1H, m, monosubstituted benzene), 8,05 (2H, m, monosubstituted benzene), 10.20 (1H, s, -NH). ^13^C-NMR (75 MHz, DMSO-*d_6_*): δ = 41.95 (CH_2_), 55.90 (CH_3_), 56.32 (CH_3_), 103.69 (Ar-C), 109.98 (Ar-C), 113.09 (Ar-C), 128.98 (Ar-C), 129.33 (Ar-C), 134.28 (Ar-C), 134.79 (Ar-C), 135.66 (Ar-C), 144.73 (Ar-C), 144.73 (Ar-C), 149.49 (Ar-C), 151.49 (Ar-C), 166.02 (Thiadiazole-C), 193.69 (C=O). HRMS (m/z): [M+H]^+^ calcd for C_16_H_17_N_3_O_3_S_2_: 388.0784; found: 388.0802.

*1.1.3.2. 2-((5-((3,4-Dimethoxyphenyl)amino)-1,3,4-thiadiazol-2-yl)thio)-1-(p-tolyl)ethan-1-one (****3b****)*

Yield: 81 % (0.365 g), purity (HPLC): >99.9%, M.P.: 157.1-158.6 ^o^C. ^1^H-NMR (300 MHz, DMSO-*d_6_*): δ = 2.40 (3H, s, -CH_3_), 3.72 (3H, s, -OCH_3_), 3.74 (3H, s, -OCH_3_), 4,90 (2H, s, -CH_2_), 6.92 (1H, d, J= 4,5 Hz trisubstituted benzene), 7.02 (1H, dd, *J_1_*= 1.5 Hz, *J_2_*= 9 Hz, trisubstituted benzene), 7.22-7.23 (1H, d, *J*= 1,5 Hz, trisubstituted benzene), 7.36-7.39 (2H, d, *J*= 4.5 Hz, disubstituted benzene), 7.94 (2H, d, *J*= 3 Hz, disubstituted benzene), 10.20 (1H, s, -NH). ^13^C-NMR (75 MHz, DMSO-*d_6_*): δ =21.71 (CH_3_), 41.90 (CH_2_), 55.95 (CH_3_), 56.32 (CH_3_), 103.70 (Ar-C), 109.98 (Ar-C), 113.10 (Ar-C), 129.11 (Ar-C), 129.86 (Ar-C), 130.23 (Ar-C), 133.17 (Ar-C), 134.80 (Ar-C), 144.74 (Ar-C), 144.83 (Ar-C), 149.49 (Ar-C), 166.01 (Thiadiazole-C), 193.22 (C=O). HRMS (m/z): [M+H]^+^ calcd for C_19_H_19_N_3_O_3_S_2_: 402.0941; found: 402.0958.

*1.1.3.3.* *2-((5-((3,4-Dimethoxyphenyl)amino)-1,3,4-thiadiazol-2-yl)thio)-1-(4-methoxyphenyl)ethan-1-one (****3c****)*

Yield: 74 % (0.347g), purity (HPLC): 98.6%, M.P.: 165.9-166.4 ^o^C. ^1^H-NMR (300 MHz, DMSO-*d_6_*): δ = 3.72 (3H, s, -OCH_3_), 3.74 (3H, s, -OCH_3_), 3.87 (3H, s, -OCH_3_), 4.87 (2H, s, -CH_2_), 6.92 (1H, d, *J*= 4.5 Hz, trisubstituted benzene), 7.02 (1H, d, trisubstituted benzene), 7.05-7.10 (2H, m, disubstituted benzene), 7.24 (1H, d, trisubstituted benzene), 8.03 (2H, d, *J*= 4.5 Hz, disubstituted benzene), 10.29 (1H, s, -NH). ^13^C-NMR (75 MHz, DMSO-*d_6_*): δ = 41.75 (CH_2_), 55.95 (CH_3_), 56.16 (CH_3_), 56.37 (CH_3_), 103.71 (Ar-C), 109.97 (Ar-C), 113.10 (Ar-C), 114.55 (Ar-C), 128.52 (Ar-C), 131.41 (Ar-C), 134.84 (Ar-C), 144.70 (Ar-C), 149.48 (Ar-C), 164.10 (Thiadiazole-C), 192.05 (C=O). HRMS (m/z):[M+H]^+^ calcd for C_19_H_19_N_3_O_4_S_2_: 418.0890; found: 418.0904.

*1.1.3.4.* *4-(2-((5-((3,4-dimethoxyphenyl)amino)-1,3,4-thiadiazol-2-yl)thio)acetyl)benzonitrile*

*(****3d****)*

Yield: 77 % (0.356 g), purity (HPLC): >99.9%, M.P.: 188.7-189.3 ^o^C. ^1^H-NMR (300 MHz, DMSO-*d_6_*): δ = 3.72 (3H, s, -OCH_3_), 3.74 (3H, s, -OCH_3_), 4.96 (2H, s, -CH_2_), 6.92 (1H, d, *J*=4.5 Hz, trisubstituted benzene), 7.03 (1H, dd, *J_1_*=1.5 Hz, *J_2_*=9 Hz, trisubstituted benzene), 7.21 (1H, d, *J*=1.5 Hz, trisubstituted benzene), 8.05-8.08 (2H, d, *J*= 4.5 Hz, disubstituted benzene), 8.17-8.20 (2H, d, *J*=4.5 Hz, disubstituted benzene), 10.21 (1H, s, -NH). ^13^C-NMR (75 MHz, DMSO-*d_6_*): δ = 41.94 (CH_2_), 55.91 (CH_3_), 56.36 (CH_3_), 103.74 (Ar-C), 110.03 (Ar-C), 113.10 (Ar-C), 116.07 (Ar-C), 118.57 (Ar-C), 129.59 (Ar-C), 133.34 (Ar-C), 134.73 (Ar-C), 138.94 (Ar-C), 144.79 (Ar-C), 149.50 (Ar-C), 166.18 (Thiadiazole-C), 193.32 (C=O). HRMS (m/z): [M+H]^+^ calcd for C_19_H_16_N_4_O_3_S_2_: 413.0737; found: 413.0753.

*1.1.3.5.* *2-((5-((3,4-Dimethoxyphenyl)amino)-1,3,4-thiadiazol-2-yl)thio)-1-(4-nitrophenyl)ethan-1-one (****3e****)*

Yield: 76 % (0.369 g), purity (HPLC): >99.9%, M.P.: 182.0-183.2 ^o^C. ^1^H-NMR (300 MHz, DMSO-*d_6_*): δ = 3.72 (3H, s, -OCH_3_), 3.74 (3H, s, -OCH_3_), 5.00 (2H, s, CH_2_), 6.92 (1H, d, *J*= 4.5 Hz, trisubstituted benzene), 7.03 (1H, dd, *J_1_*=1.5 Hz, *J_2_*=9 Hz, trisubstituted benzene), 7.20-7.21 (1H, d, *J*=1.5 Hz, trisubstituted benzene), 8.26-8.29 (2H, d, *J*=4.5 Hz, disubstituted benzene), 8.37-8.40 (2H, d, *J*=4.5 Hz, disubstituted benzene), 10.21 (1H, s, -NH).^13^C-NMR (75 MHz, DMSO-*d_6_*): δ = 42.11 (CH_2_), 55.91 (CH_3_), 56.36 (CH_3_), 103.75 (Ar-C), 110.04 (Ar-C), 113.09 (Ar-C), 124.38 (Ar-C), 130.42 (Ar-C), 134.74 (Ar-C), 140.39 (Ar-C), 144.79 (Ar-C), 149.49 (Ar-C), 150.66 (Ar-C), 166.18 (Thiadiazole-C), 193.14 (C=O). HRMS (m/z): [M+H]^+^ calcd for C_18_H_16_N_4_O_5_S_2_: 433.0635; found: 433.0658.

*1.1.3.6. 1-(4-Chlorophenyl)-2-((5-((3,4-dimethoxyphenyl)amino)-1,3,4-thiadiazol-2-yl)thio)ethan-1-one (****3f****)*

Yield: 76 % (0.359 g), purity (HPLC): >99.9%, M.P.: 162.2-163.3 ^o^C. ^1^H-NMR (300 MHz, DMSO-*d_6_*): δ = 3.72 (3H, s, -OCH_3_), 3.74 (3H, s, -OCH_3_), 4.93 (2H, s, CH_2_), 6.92 (1H, d, *J*=4.5 Hz, trisubstituted benzene), 7.03 (1H, dd, *J_1_*=1.5 Hz, *J_2_*=9 Hz, trisubstituted benzene), 7.22 (1H, d, *J*=1.5 Hz, trisubstituted benzene), 7.65 (2H, d, *J*= 3 Hz, disubstituted benzene), 8.04-8.07 (2H, d, *J*=4.5 Hz, disubstituted benzene), 10.20 (1H, s, -NH). ^13^C-NMR (75 MHz, DMSO-*d_6_*): δ = 41.84 (CH_2_), 55.95 (CH_3_), 56.36 (CH_3_), 103.73 (Ar-C), 110.01 (Ar-C), 113.10 (Ar-C), 129.44 (Ar-C), 130.91 (Ar-C), 134.38 (Ar-C), 134.77 (Ar-C), 139.20 (Ar-C), 144.77 (Ar-C), 149.50 (Ar-C), 151.28 (Ar-C), 166.10 (Thiadiazole-C), 192.84 (C=O). HRMS (m/z): [M+H]^+^ calcd for C_18_H_16_N_3_O_3_S_2_Cl: 422.0394; found: 422.0397.

*1.1.3.7. 2-((5-((3,4-Dimethoxyphenyl)amino)-1,3,4-thiadiazol-2-yl)thio)-1-(4-fluorophenyl)ethan-1-one (****3g****)*

Yield: 80 % (0.364 g), purity (HPLC): >99.9%, M.P.: 171.1-171.8 ^o^C. ^1^H-NMR (300 MHz, DMSO-*d_6_*): δ = 3.72 (3H, s, -OCH_3_), 3.75 (3H, s, -OCH_3_), 4.93 (2H, s, CH_2_), 6.92 (1H, d, J=3 Hz, trisubstituted benzene), 7.03 (1H, dd, *J_1_*=1.5 Hz, *J_2_*=9 Hz, trisubstituted benzene), 7.22 (1H, d, trisubstituted benzene), 7.38-7.44 (2H, t, *J*=4.5 Hz, disubstituted benzene), 8.11-8.16 (2H, dd, *J_1_*=3 Hz, *J_2_*=4.5 Hz, disubstituted benzene), 10.20 (1H, s, -NH). ^13^C-NMR (75 MHz, DMSO-*d_6_*): δ = 41.85 (CH_2_), 55.94 (CH_3_), 56.36 (CH_3_), 103.72 (Ar-C), 110.00 (Ar-C), 113.10 (Ar-C), 116.24 (Ar-C), 116.53 (Ar-C), 132.02 (Ar-C), 132.14 (Ar-C), 134.77 (Ar-C), 144.76 (Ar-C), 149.50 (Ar-C), 164.14 (Ar-C), 166.08 (Thiazole-C), 167.49 (Ar-C), 192.38 (C=O). HRMS (m/z): [M+H]^+^ calcd for C_18_H_16_N_3_O_3_FS_2_: 406.0690; found: 406.0707.

*1.1.3.8. 1-(4-Bromophenyl)-2-((5-((3,4-dimethoxyphenyl)amino)-1,3,4-thiadiazol-2-yl)thio)ethan-1-one (****3h****)*

Yield: 77 % (0.402 g), purity (HPLC): 98.3%, M.P.: 178.5-179.0 ^o^C. ^1^H-NMR (300 MHz, DMSO-*d_6_*): δ = 3.72 (3H, s, -OCH_3_), 3.75 (3H, s, -OCH_3_), 4.92 (2H, s, CH_2_), 6.92 (1H, d, *J*=3 Hz trisubstituted benzene), 7.01-7.04 (1H, m, trisubstituted benzene), 7.21-7.22 (1H, d, *J*=1.5 Hz, trisubstituted benzene), 7.78-7.81 (2H, d, *J*=4.5 Hz, disubstituted benzene), 7.96-7.99 (2H, d, *J*=4.5 Hz, disubstituted benzene), 10.20 (1H, s, -NH). ^13^C-NMR (75 MHz, DMSO-*d_6_*): δ = 41.82 (CH_2_), 55.94 (CH_3_), 56.35 (CH_3_), 103.72 (Ar-C), 110.00 (Ar-C), 113.09 (Ar-C), 128.43 (Ar-C), 130.98 (Ar-C), 132.40 (Ar-C), 134.69 (Ar-C), 134.76 (Ar-C), 144.76 (Ar-C), 149.49 (Ar-C), 151.27 (Ar-C), 166.09 (Thiadiazole-C), 193.06 (C=O). HRMS (m/z): [M+H]^+^ calcd for C_18_H_16_N_3_O_3_S_2_Br: 465.9889; found: 465.9887.

*1.1.3.9.* *1-(2,4-Dichlorophenyl)-2-((5-((3,4-dimethoxyphenyl)amino)-1,3,4-thiadiazol-2-yl)thio)ethan-1-one (****3i****)*

Yield: 79 % (0.404 g), purity (HPLC): 96.4%, M.P.: 139.5-141.8 ^o^C. ^1^H-NMR (300 MHz, DMSO-*d_6_*): δ = 3.73 (3H, s, -OCH_3_), 3.76 (3H, s, -OCH_3_), 4.78 (2H, s, CH_2_), 6.93 (1H, d, *J*=3 Hz, trisubstituted benzene), 7.03-7.06 (1H, m, trisubstituted benzene), 7.20-7.21 (1H, d, *J*=1.5 Hz, trisubstituted benzene), 7.59-7.63(1H, dd, *J_1_*=1.5 Hz, *J_2_*=9 Hz, trisubstituted benzene) 7.78-7.79 (1H, d, *J*=1.5 Hz, trisubstituted benzene), 7.87 (1H, d, *J*=3 Hz, trisubstituted benzene), 10.21 (1H, s, -NH). ^13^C-NMR (75 MHz, DMSO-*d_6_*): δ = 44.00 (CH_2_), 55.95 (CH_3_), 56.36 (CH_3_), 103.75 (Ar-C), 110.05 (Ar-C), 113.09 (Ar-C), 128.05 (Ar-C), 130.67 (Ar-C), 131.93 (Ar-C), 132.23 (Ar-C), 134.74 (Ar-C), 135.64 (Ar-C), 137.32 (Ar-C), 144.80 (Ar-C), 149.50 (Ar-C), 150.91 (Ar-C), 166.17 (Thiadiazole-C), 194.86 (C=O). HRMS (m/z): [M+H]^+^ calcd for C_18_H_15_N_3_O_3_S_2_Cl_2_: 456.0005; found:456.0000.

*1.1.3.10.* *1-(3,4-Dichlorophenyl)-2-((5-((3,4-dimethoxyphenyl)amino)-1,3,4-thiadiazol-2-yl)thio)ethan-1-one (****3j****)*

Yield: 74 % (0.378 g), purity (HPLC): >99.9%, M.P.: 176.4-177.1 ^o^C. ^1^H-NMR (300 MHz, DMSO-*d_6_*): δ = 3.72 (3H, s, -OCH_3_), 3.75 (3H, s, -OCH_3_), 4.93 (2H, s, CH_2_), 6.91-6.94 (1H, d, *J*=4.5 Hz, trisubstituted benzene), 7.01-7.04 (1H, m, trisubstituted benzene), 7.21-7.22 (1H, d, *J*=1.5 Hz, trisubstituted benzene), 7.84-7.87 (1H, d, *J*=4.5 Hz, trisubstituted benzene) 7.97-8.01 (1H, dd, *J_1_*=1.5 Hz, *J_2_*=9 Hz, trisubstituted benzene), 8.27 (1H, d, trisubstituted benzene), 10.21 (1H, s, -NH). ^13^C-NMR (75 MHz, DMSO-*d_6_*): δ = 41.78 (CH_2_), 54.94 (CH_3_), 55.35 (CH_3_), 56.31 (CH_3_), 57.26 (CH_3_), 103.69 (Ar-C), 109.99 (Ar-C), 111.99 (Ar-C), 113.05 (Ar-C), 114.11 (Ar-C), 127.81 (Ar-C), 127.91 (Ar-C), 128.96 (Ar-C), 129.78 (Ar-C), 130.54 (Ar-C), 130.95 (Ar-C), 131.68 (Ar-C), 132.11 (Ar-C), 132.81 (Ar-C), 135.85 (Ar-C), 149.47 (Ar-C), 166.20 (Thiadiazole-C), 192.17 (C=O). HRMS (m/z): [M+H]^+^ calcd for C_18_H_15_N_3_O_3_S_2_Cl: 456.0005; found: 456.0003.

**Spectra 1.** ^1^H-NMR spectra of compound **3a**

**Spectra 2.** ^13^C-NMR spectra of compound **3a**

**Spectra 3.** HRMS spectra of compound **3a**

**Spectra 4.** ^1^H-NMR spectra of compound **3b**

**Spectra 5.** ^13^C-NMR spectra of compound **3b**

**Spectra 6.** HRMS spectra of compound **3b**

**Spectra 7.** ^1^H-NMR spectra of compound **3c**

**Spectra 8.** ^13^C-NMR spectra of compound **3c**

**Spectra 9.** HRMS spectra of compound **3c**

**Spectra 10.** ^1^H-NMR spectra of compound **3d**

**Spectra 11.** ^13^C-NMR spectra of compound **3d**

**Spectra 12.** HRMS spectra of compound **3d**

**Spectra 13.** ^1^H-NMR spectra of compound **3e**

**Spectra 14.** ^13^C-NMR spectra of compound **3e**

**Spectra 15.** HRMS spectra of compound **3e**

**Spectra 16.** ^1^H-NMR spectra of compound **3f**

**Spectra 17.** ^13^C-NMR spectra of compound **3f**

**Spectra 18.** HRMS spectra of compound **3f**

**Spectra 19.** ^1^H-NMR spectra of compound **3g**

**Spectra 20.** ^13^C-NMR spectra of compound **3g**

**Spectra 21.** HRMS spectra of compound **3g**

**Spectra 22.** ^1^H-NMR spectra of compound **3h**

**Spectra 23.** ^13^C-NMR spectra of compound **3h**

**Spectra 24.** HRMS spectra of compound **3h**

**Spectra 25.** ^1^H-NMR spectra of compound **3i**

**Spectra 26.** ^13^C-NMR spectra of compound **3i**

**Spectra 27.** HRMS spectra of compound **3i**

**Spectra 28.** ^1^H-NMR spectra of compound **3j**

**Spectra 29.** ^13^C-NMR spectra of compound **3j**

**Spectra 30.** HRMS spectra of compound **3j**

**Chromatogram** **1.** HPLC chromatogram of compound **3a**

**Chromatogram** **2.** HPLC chromatogram of compound **3b**

**Chromatogram** **3.** HPLC chromatogram of compound **3c**

**Chromatogram** **4.** HPLC chromatogram of compound **3d**

**Chromatogram** **5.** HPLC chromatogram of compound **3e**

**Chromatogram** **6.** HPLC chromatogram of compound **3f**

**Chromatogram** **7.** HPLC chromatogram of compound **3g**

**Chromatogram** **8.** HPLC chromatogram of compound **3h**

**Chromatogram** **9.** HPLC chromatogram of compound **3i**

**Chromatogram** **10.** HPLC chromatogram of compound **3j**
